# A Method to Find Longevity-Selected Positions in the Mammalian Proteome

**DOI:** 10.1371/journal.pone.0038595

**Published:** 2012-06-11

**Authors:** Jeremy Semeiks, Nick V. Grishin

**Affiliations:** 1 Molecular Biophysics Program and Medical Scientist Training Program, University of Texas Southwestern Medical Center, Dallas, Texas, United States of America; 2 Department of Biochemistry and Howard Hughes Medical Institute, University of Texas Southwestern Medical Center, Dallas, Texas, United States of America; University of South Florida College of Medicine, United States of America

## Abstract

Evolutionary theory suggests that the force of natural selection decreases with age. To explore the extent to which this prediction directly affects protein structure and function, we used multiple regression to find longevity-selected positions, defined as the columns of a sequence alignment conserved in long-lived but not short-lived mammal species. We analyzed 7,590 orthologous protein families in 33 mammalian species, accounting for body mass, phylogeny, and species-specific mutation rate. Overall, we found that the number of longevity-selected positions in the mammalian proteome is much higher than would be expected by chance. Further, these positions are enriched in domains of several proteins that interact with one another in inflammation and other aging-related processes, as well as in organismal development. We present as an example the kinase domain of anti-Müllerian hormone type-2 receptor (AMHR2). AMHR2 inhibits ovarian follicle recruitment and growth, and a homology model of the kinase domain shows that its longevity-selected positions cluster near a SNP associated with delayed human menopause. Distinct from its canonical role in development, this region of AMHR2 may function to regulate the protein’s activity in a lifespan-specific manner.

## Introduction

Despite a 10–100-fold difference in maximum lifespan (MLS), most known mammal species show similar phenotypes of aging [1]. This observation suggests that the genetic determinants of mammalian aging and lifespan may be relatively plastic. The classical evolutionary theory of antagonistic pleiotropy [2] posits that aging is an effect of the decrease in selection pressure that occurs after successful reproduction. Conversely, lifespan extension has been shown to occur when selection pressure increases in later age [3]. Still relatively unexplored are the specific molecular mechanisms that determine differences in mammalian lifespan. Many mechanisms are possible and likely occur simultaneously, including changes in the sequence, structure, function, and expression of RNA and proteins.

Here, we focus on changes in proteins caused by fixed substitutions. In this context, two recent studies [4], [5] predicted a simple consequence of the evolutionary theory: we might expect that proteins necessary for long mammalian lifespan would have fewer substitutions, i.e. show more conservation, in long-lived versus short-lived species. Thus, it may be possible to identify aging-related proteins (more specifically, families of orthologous proteins) by inferring and comparing some measure of such preferential substitutions, here called ";longevity-selected positions";, among the several dozen mammal species whose proteomes are available. In particular, Jobson et al. [4] accomplished this from the perspective of classical genetics, applying to a codon model a measure similar to the well-known dN/dS ratio (reviewed in [6]). Two relevant features of their method were that (1) species were binned as ";long-lived"; or ";short-lived"; based on MLS and (2) in keeping with the conventional framework, the total estimate of synonymous substitutions in a gene determined the threshold for whether any particular codon position in that gene was called longevity-selected.

In contrast, we start from the perspective of protein structure, which emphasizes two concepts that may be useful to find interesting longevity-selected positions. First, positions within a protein are not interchangeable; whatever the estimated synonymous substitution rate, a single nonsynonymous substitution in a certain structural context can change a protein’s function. Second, not all nonsynonymous substitutions are equal; rather, a priori we should expect those amino acid substitutions that commonly change biochemical function to matter most for the function of a specific protein. Based on these observations, we present a simple regression-based method to find longevity-selected positions in orthologous protein families of mammals. Our method employs the phylogenetic generalized least squares framework (PGLS, equivalent to phylogenetic independent contrasts; [7]) to relate, for each position (column) of a protein alignment, the MLS of the species represented in the column to the biochemical divergence of their residues from the residue of a long-lived reference species. Two benefits of PGLS are that (1) it is straightforward to control for common gerontological confounders, including species-specific mutation rate, body mass, and shared phylogeny [8], and (2) we can naturally fit a continuous variable such as MLS, removing the need to arbitrarily bin species.

We use our method as a starting point to analyze longevity-selected positions, placing emphasis on their structural contexts. Our results concern both the proteome as a whole and a specific protein domain identified by our analysis, the kinase domain of anti-Müllerian hormone type-2 receptor (AMHR2; kinase nomenclature per [9]). AMHR2 is a receptor protein serine/threonine kinase in the TGF-beta Receptor Type II (TGFBR2) subfamily. The canonical role of AMHR2 is to inhibit the Müllerian ducts during development of the male fetus, and mutations cause the rare disease persistent Müllerian duct syndrome (PMDS; [10]). More recently, a role for AMHR2 in ovarian follicle development of the adult female has also been identified [11], and this noncanonical function may be relevant to our findings.

## Materials and Methods

### 2.1. Selection of Positions to Analyze

We analyzed a selected subset of the OrthoMaM database version 6, which comprises multiple sequence alignments (MSAs) of 11,746 protein ortholog families from 36 mammalian species [12]. Most of the sequences in the database were extracted from low-coverage genomes, so completeness and quality of alignments varied considerably. We first masked nonstandard isoforms and other divergent subsequences using a sliding window-based approach. Specifically, we excluded from further analysis any subsequence of at least 10 residues in which every 10-residue window had at least four residues each with less than 30% sequence identity to the rest of its column. We also excluded the three non-eutherian species due to high sequence divergence. Of the remaining data, we selected for fitting only columns that (1) included at least ten characters overall and (2) specifically included characters for both human and shrew. We refer to this subset as *selected columns*.

### 2.2. Column Correction, Fitting, and Analysis

We define *fit columns* as the subset of selected columns that have at least three characters different from the human reference character, and *conserved columns* as all other selected columns. We independently fit each column in the *fit* subset to a phylogenetic generalized linear model [7], as implemented in the R package caper, version 0.4 [13]. Briefly, this framework assumes a Brownian model of trait evolution and uses the method of generalized least squares to perform multiple regression with correction for global phylogenetic dependence, as indicated by the mammalian supertree. Specifically, for each column we fit the regression model.<$>\raster="rg1"<$>

Here, *MLS* are the maximum lifespans and *mass* the body masses of each species as reported in AnAge version 11 [14]. *B80_mut_* are the BLOSUM80 scores for each nonhuman ortholog character in the column versus the human ortholog character [15], corrected for mutation rate as described below. We used caper’s default parameter values, including fixed nonterminal branch length multiplier (λ) of 1 for phylogenetic correction. As the input phylogeny, we used the mammalian supertree [16], which is ultrametric. Fitting completed after running for one day on a standard single processor (2.2 GHz, 16 GB RAM).

To control for each species’ overall mutation rate, we used an approach similar to that of Li and de Magalhães [5]. Specifically, to estimate these mutation rates we constructed by maximum likelihood (protml, [17]) a tree whose branch length was the expected number of amino acid substitutions over all fit columns. We used this tree to correct each BLOSUM80 score for the mutation rate of its respective species *s* relative to human by adding log_10_(*m_s_*/*m_ref_*) to the score, where *m_s_* was the total tree length of species *s* and *m_ref_* was the tree length of human. We restricted the range of each B80_mut_ score to the standard range of B80 scores attainable by its human character.

Each fit yielded both a longevity-selected slope, *b_MLS_*, and p-value, *p_MLS_.* We defined as a *longevity-selected position* any column with both *b_MLS_*>0 and *p_MLS_*<0.01. Conserved columns were assigned *b_MLS_=*0 and *p_MLS_*=1.

For the large-scale analyses, in the human orthologs we predicted secondary structure with PSIPRED [18]; differences in composition were tested with Pearson’s χ^2^ test. Protein domain definitions and other features were taken from Swiss-Prot [19] and mapped to OrthoMaM alignments by aligning each Swiss-Prot sequence to its human counterpart in OrthoMaM. Ontology enrichment analysis was performed using the Functional Annotation Clustering module of DAVID [20] with default parameters, including the human genome as background. We report only Benjamini-corrected p-values. For the rolling-window analysis of positions, we included only contiguous blocks of 10 selected positions.

To construct the randomized control dataset, for each species *A* except human, we swapped MLS, body mass, and phylogenetic label with those of one species *B*, which was randomly selected without replacement. All alignments remained unchanged, meaning that the same set of columns were fit in both the randomized control and real sets.

### 2.3. Homology Modeling and Structural Analysis of AMHR2

We created a homology model of AMHR2 with Modeler [21], using as template the kinase domain of BMPR2 (PDB ID: 3G2F). Homology models made with SWISS-MODEL [22], or using as template the kinase domain of ACVR2B (PDB ID: 2QLU), yielded similar results. Positional conservation was calculated with AL2CO [23], using 3G2F as the structural model.

## Results and Discussion

### 3.1. Due to Overall Conservation, Most Positions in the Mammalian Proteome are Not Longevity-selected

**Figure 1:**
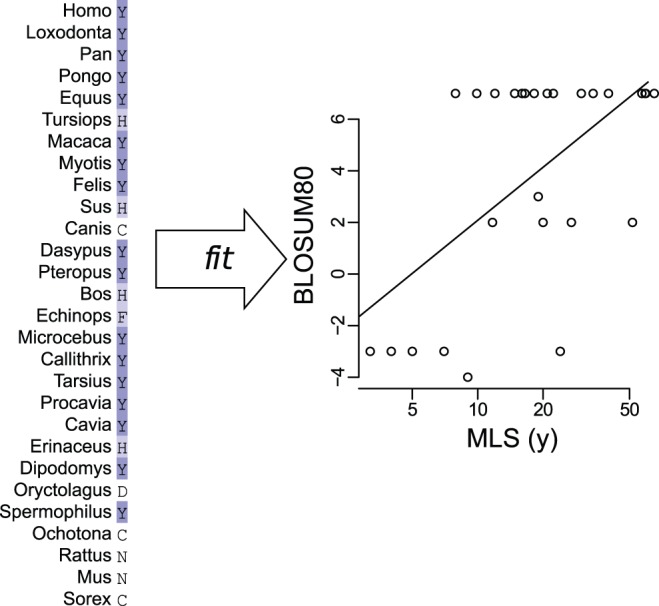
Conceptual example of our multiple regression method applied to a single column (Y465 of AMHR2; full result in Table S2). (left) Characters shown ordered by species MLS. For each non-human species, we calculate the similarity score (";BLOSUM80";) for the species’ amino acid character versus the human character (here Y); e.g., this score for Tursiops would be the similarity score for H versus Y, which is 2. (right) We then fit the MLS of all non-human species to their similarity scores; e.g., Tursiops’ contribution to this fit is the point (52, 2). Not shown are the steps to correct for mutation rate and shared phylogeny, and the simultaneous fit of body mass. For this column, the data provide relatively strong support for a nonzero slope in the fit of similarity to MLS, even given trends in mutation rate, phylogeny, and body mass, and so this position is assigned a relatively significant p-value (*p_MLS_*<0.01).

To identify specific positions (i.e., alignment columns) in the mammalian proteome that are conserved in long-lived but not in short-lived species, we fit a generalized multiple regression model to each position independently. Our overall approach followed from the observation that many of the positions we sought were distinguished by high correlation between (1) each species’ MLS and (2) functional similarity of each species’ residue to that of a long-lived reference species. We used human as the reference species, both because it was the longest-lived mammal whose sequence was available and because we had the best confidence in the accuracy of its sequence. Figure 1 shows a simplified conceptual example of our approach. Our method also accounted for each species’s body mass and overall mutation rate relative to human. We emphasize that we chose multiple regression not for rigorous statistical reasons, but only as a computational tool to help form new biological hypotheses.

**Figure 2:**
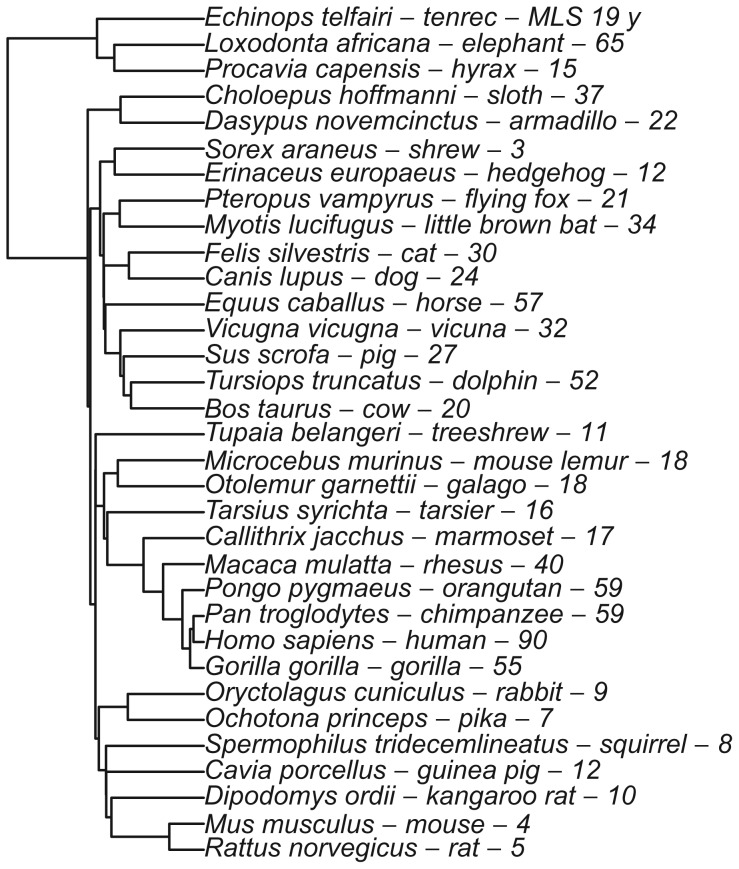
Phylogeny of species used in this study. Shown next to each species’ node are its binomial, common name, and MLS.

In this manner, we fit selected columns among the proteomes of 33 species (Figure 2), with MLS ranging from 3 y (shrew) to 90 y (human). Initially, we attempted to fit the entire unfiltered OrthomMaM database. However, this effort yielded many obvious false-positives driven by low sample size and data of questionable quality, including highly divergent sequence at exon-intron boundaries and alternate isoforms. For this reason, we implemented several heuristic filters for selection. In particular, because rodents are over-represented among the short-lived species of OrthoMaM, we selected only columns containing a character for shrew (*Sorex araneus)*, the shortest-lived non-rodent. (Sections 2.1 and 2.2 give precise definitions of the sets *selected, fit,* and *conserved.*) Using these criteria, we verified that most selected positions in the mammalian proteome (80%) are conserved across all species (Table 1; also found previously [4], [5]). In particular, at least 10 of 261 genes in the GenAge database of aging-related genes [24] have protein products that show near-complete conservation in mammals (≥90% ratio of conserved positions to total human ortholog length), for example beta-catenin *(CTNNB1)*, valosin-containing protein *(VCP)*, fibroblast growth factor receptor 1 *(FGFR1)*, and lamin A *(LMNA)*. Thus, if these genes contribute to differences in mammalian longevity, it is likely via some mechanism other than structural differences in their protein products.

**Table 1:** Number of positions and alignments selected, fit, and conserved, as defined in Sections 2.1 and 2.2.

subset	columns (×10^6^)	alignments
include human	7.01	12,746^a^
–selected	3.64	7,708^a^
––fit	0.73	7,590^a^
––conserved	2.91	118^b^

a Number of alignments that include at least one position in the indicated subset.

b Number of alignments that include only conserved positions.

### 3.2. Among Nonconserved Positions, Longevity-selected Positions Occur More often than Expected by Chance

For each selected position, our fitting procedure yielded both a longevity-associated slope *(b_MLS_)* and associated p-value *(p_MLS_),* as well as corresponding measures for body mass (*b_mass_* and *p_mass_*). Only those positions with significantly positive slope were called *longevity-selected* (*b_MLS_*>0 and *p_MLS_*<0.01) or *mass-selected* (*b_mass_*>0 and *p_mass_*<0.01). There were no positions that were both longevity-selected and mass-selected, suggesting that *p_MLS_<*0.01 was a reasonable cutoff in general for our analyses.

**Figure 3:**
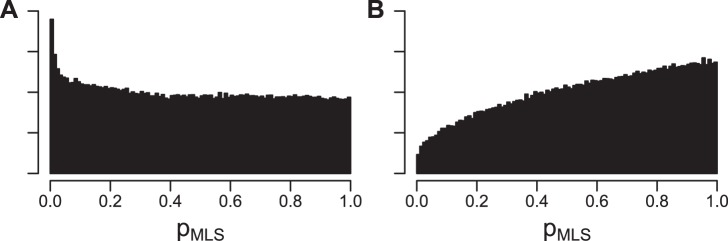
Density histograms of *p_MLS_* values. A. Real data. B. Randomized control. Each histogram shows the n=734,741 fit positions with *b_MLS_*>0. See Section 3.2 for details.

Among positions with positive MLS slope, we analyzed the distribution of p-values in order to determine whether proteome-wide trends existed with regard to longevity-selected positions (Figure 3). As a negative control, we also analyzed a matched set of positions whose MLS, body mass, and phylogenetic position had been randomly swapped (";randomized control";). If there were no overall relationship between MLS and amino acid conservation, then we would expect significant p-values to be no more common than nonsignificant p-values after accounting for shared phylogeny and body mass. This is indeed the case for the randomized control (Figure 3B). However, for the real data (Figure 3A), positions with more significant p-values are clearly overrepresented relative to those with less significant p-values. These results indicate that overall, longevity-selected positions in the mammalian proteome are much more likely than would be expected by chance.

This finding is robust to several perturbations of the data (Figure S1), including use of chimp as the reference instead of human (Figure S1A) and use of BLOSUM62 scores instead of BLOSUM80 scores (Figures S1D and S1E). We note that although BLOSUM62 is more commonly used than BLOSUM80, in this case BLOSUM80 is more appropriate because mammalian proteomes typically have 80–90% sequence identity. The result also holds for input in which all nonhuman primates except one (here, rhesus) are omitted (Figure S1B), indicating that it is not an artifact of primate over-representation in OrthoMaM. Almost all (7,689/7,723) of the positions called significant in Figure 3A have *p_MLS_<*0.05 in this control set, suggesting that its relative lack of very significant *p_MLS_* values is simply due to fewer data available for each position (n=26 versus 32 species available to fit). Finally, as we would expect the result does not hold when dog, a shorter-lived species, is used as the reference instead of human (Figure S1C).

A plausible biological hypothesis to explain this overall plethora of longevity-selected positions is that the evolution of longer mammalian lifespan requires particular concerted patterns of substitutions throughout the proteome that subtly affect protein properties such as binding affinity, folding, and stability. Consistent with this hypothesis is that relative to mouse (MLS 4 y), the proteome of naked mole rat (a rodent with MLS ∼30 y) is more resistant to urea-induced unfolding [25], suggesting increased protein stability in the longer-lived rodent. An analogous process requiring concerted patterns of substitution may be the convergent evolution of hyperthermostability in archaea and bacteria [26].

To determine overall trends in longevity-selected positions with regard to structural features of the proteome, we created and searched databases of both predicted secondary structure and predicted disordered regions for the human proteome. Table 2 shows the secondary structure composition of the human genome as a whole, as well as in the longevity-selected positions of both the real fit data and the randomized control data. This table shows two trends. The first trend is that, in both real and randomized longevity-selected positions, random coils are overrepresented, at the expense of α-helices and β-strands, relative to the human proteome as a whole (χ^2^(2, n=7,702)=265.23, *p*<2.2e-16). This is easily explained by the observation that sequence in random coils tends to be less conserved than other sequence; thus, fit positions will tend to be overrepresented in these regions.

**Table 2:** Predicted secondary structure in all positions of the human proteome and in two subsets of longevity-selected positions.

	α-helix	β-strand	random coil	total
human proteome	2,108,348 (29.95%)	956,422 (13.59%)	3,974,432 (56.46%)	7,039,202 (100%)
real data	1,922 (24.95%)	736 (9.56%)	5,044 (65.49%)	7,702 (100%)
randomized control	160 (23.26%)	62 (9.01%)	466 (67.73%)	688 (100%)

The second trend is that, relative to randomized longevity-selected positions, real longevity-selected positions are slightly enriched in α-helices at the expense of β-strands (χ^2^(2, n=7,702)=18.36, *p*=1.0e-4). The significance of this finding is unknown, but it is possible that an abundance of α-helices imparts extra stability to proteins and protein complexes, e.g. via coiled-coil interactions [27].

### 3.3. Longevity-selected Positions are Enriched in Protein Domains with Known Roles in Inflammation, Development, and other Diverse Functions

The majority of longevity-selected positions are located in regions of proteins that are unannotated and presumably unstructured. Since these regions are generally of unknown function at present, it is difficult to interpret the biochemical significance of substitutions within them. Thus, to find longevity-selected positions with the best likelihood of causing well-characterized changes to protein structure and function, we next narrowed our focus to known protein domains. Specifically, we compiled a list of all 129 domains that contain at least two longevity-selected positions. The 129 domains are contained in 114 proteins. Table S1 summarizes the proteins and domains, and Table S2 shows data for each longevity-selected position in the domains, including number of characters (i.e., species) fit and all slopes and p-values. Five genes for the 114 proteins shown in these tables are present in the GenAge database [24]: serine-protein kinase ATM, ATM; serine/threonine protein kinase ATR, ATR; breast cancer type 1 susceptibility protein, BRCA1; ATP-dependent DNA helicase Q4, RECQL4; and DNA-dependent protein kinase catalytic subunit, PRKDC. Functional annotation clustering revealed that several of the 114 proteins belong to functional classes that have been associated with aging (Table 3). We give three examples. First, leukemia inhibitory factor receptor (LIFR) and interleukin-6 receptor subunit beta (IL6ST) dimerize to form the receptor for leukemia inhibitory factor (LIF; not present in our results), whose signaling is upregulated with age in association with thymic atrophy [28]. Second, arachidonate 15-lipoxygenase (ALOX15) is an enzyme that is upregulated in aging rat brain [29]; it functions in production of inflammatory leukotrienes, and may also function nonenzymatically to upregulate NFκB [30]. Third, several of these proteins function in blood coagulation, markers of which increase with age and may interact with markers of inflammation [31]. Detailed structural analysis of these domains remains to be performed.

**Table 3:** Selected clusters, not mutually exclusive, of ontology terms enriched in top protein domains.

functional class	*p*	examples
extracellular region	5.1e-5	BTD, CP, LAMA2, LAMA3, PRSS12
cytokine-mediated signaling pathway	0.033	JAK1, IL31RA, IL6ST, LIFR, RIKP1, KIT
protein tyrosine kinase activity	0.018	JAK1, ROS1, MST1R, NIN, OBSCN, KIT, TYK2
multicopper oxidase; copper ion binding	9.4e-3	AFP, CP, F8, HEPHL1
developmental process	0.041	AFP, AMHR2, ALOX15, CFTR, ATR, ATM, BRCA1, RECQL4
motor activity; myosin complex	0.015	KIF18B, KIF20B, KIF22, MYO5C, MYO7B, MYO18A
complement and coagulation cascades;humoral immune response	0.061	F8, F11, CR2, C1R, CFD, LTF, CD83
serine-type endopeptidase activity	0.063	F11, C1R, CFD, KLK6, LTF, PRSS12

Table 3 also indicates that several of our hits are involved in development, and this highlights a limitation of our data set. It is well-known that developmental schedule and longevity have co-evolved in mammals [1], [32]; thus, these positions may specifically be conserved due to their effect on development, or they may pleiotropically affect both development and adult longevity. We did not attempt to correct for developmental schedule when fitting the data. In our framework, such correction is possible in concept by adding to the regression model a third predictor variable, species age at maturity. However, in the present set of species, we face the problem of multicollinearity: age at maturity is too well-correlated with MLS for correction to be a realistic goal (R^2^=0.48 on n=30 species for age at female maturity after phylogenetic correction). Additionally, no maturity data are available for three important species in our set: *Pteropus, Tarsius,* and *Tupaia.* Thus, it is not possible to distinguish in general whether the positions found by our method are conserved due to their roles in development, adult longevity, or both. But most of these proteins, including our next example of AMHR2, do have verified roles in the adult organism and may plausibly affect longevity.

### 3.4. Longevity-selected Positions Cluster in the Kinase Domain and C-terminal Tail of AMHR2

The domain containing the greatest number of longevity-selected positions (n=7) was the protein kinase domain of AMHR2, a protein introduced in Section 1. In human, this domain comprises residues 203–517. Downstream of this domain is the C-terminal tail of the protein, residues 518–573. This cysteine-rich tail is unique to the AMHR2 ortholog family and is predicted to lack secondary structure. On average, the residues in the region 463–573 that do not form secondary structure have *p_MLS_* values that are consistently in the top 2% of the 3.6 million selected positions (median *p_MLS_*<0.568 by sliding window analysis). But this trend does not hold for AMHR2 overall (median *p_MLS_* is 0.800 excluding secondary structure positions), suggesting that specifically the kinase C-lobe and downstream C-terminal tail of AMHR2 are under lifespan-related selective pressure.

**Figure 4:**
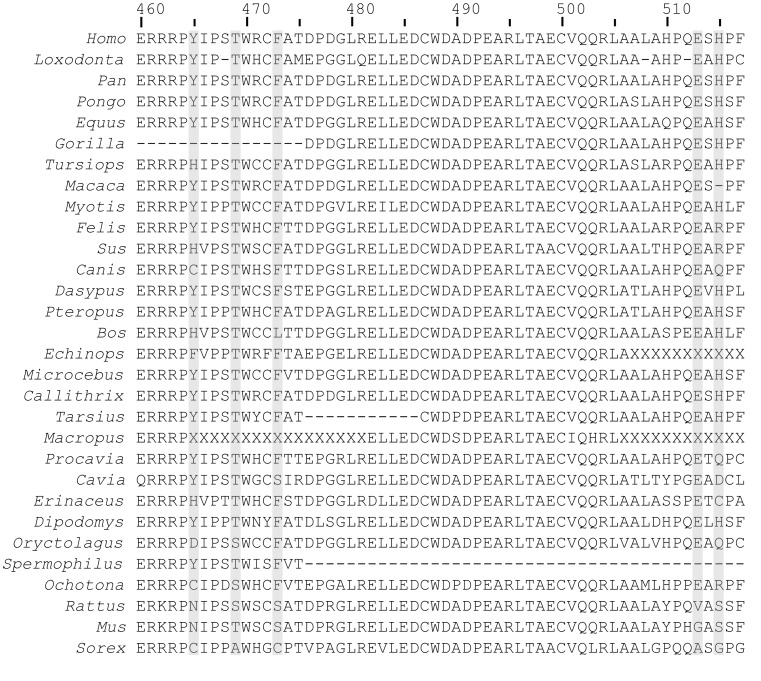
OrthoMaM alignment of the C-terminal regions of AMHR2 ortholog kinase domains. Orthologs are ordered by species MLS. The five longevity-selected positions in this region (Y465, T469, F473, E513, and H515) are highlighted in gray. ";X"; indicates regions that we masked due to excessive divergence (Section 2.1). Long regions of gaps are not necessarily real genome deletions, but are more likely to have been missed during genome assembly or annotation.

**Figure 5:**
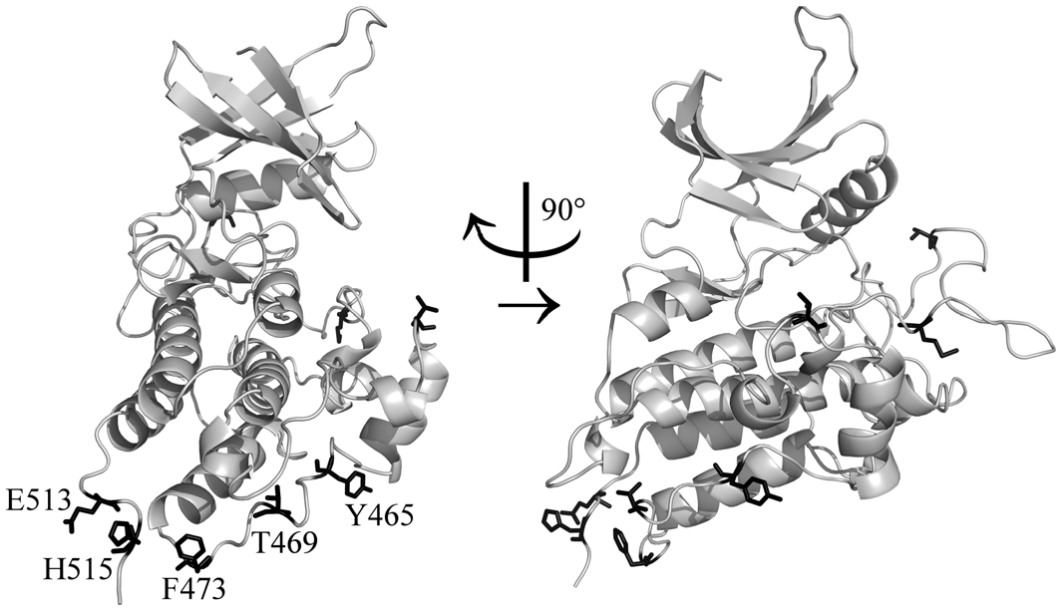
AMHR2 kinase domain mapped onto experimental structure of BMPR2 kinase domain. All eight longevity-selected positions found in this domain are shown as black sticks and are further described in Table S2. The five longevity-selected positions discussed in the text are labeled; they are found on the αG–αH loop (Y465, T469, and F473) and the C-terminal loop following αI (E513 and H515).

In sequence, five of the eight longevity-selected positions in the kinase domain of AMHR2 concentrate in two regions near the C-terminus of the domain (Figure 4). To determine the likely locations of our longevity-selected positions on the structure of AMHR2, we mapped them to the recently-solved structure of the kinase domain of bone morphogenetic protein type 2 receptor (BMPR2). This domain is the closest homolog to the kinase domain of AMHR2, with 40% sequence identity. Our mapping (Figure 5) shows that these five longevity-selected positions cluster at or near a common surface of the AMHR2 kinase C-lobe. Specifically, they are all located on two loops near the bottom-front face of the domain: the αG–αH loop (Y465, T469, and F473) and the C-terminal loop following αI (E513 and H515). All five side-chains at least partially face solvent.

The side-chain of Y465 forms an intra-loop hydrogen bond with R462, a conserved residue. Thus, in conjunction with P464, the length of the Y465 side-chain constrains the angle of a second conserved arginine, R463, which forms hydrogen bonds to residues on αF and the αF-αG loop. Y465 is conserved in 8/9 species with MLS of at least 30 y, but in 9/19 shorter-lived species it is instead H, F, C, N, or D, none of which (except possibly H) can hydrogen-bond with R463 at the same angle as can Y. We predict that these substitutions would abolish at least the hydrogen bond with R462, destabilizing the αG–αH loop. Although the αG–αH and C-terminal loops are both quite divergent overall and contain several positions that did not meet our significance criterion for longevity conservation, Y465 is the only nonconserved residue within them whose side-chain is predicted to interact with another residue of AMHR2.

T469 represents a position that may be differentially phosphorylated, but it also highlights some current limitations of our method and data set. T469 is the only predicted phosphorylation site on the αG–αH loop (NetPhos score 0.692; [33]). However, Figure 4 shows that this residue is consistently serine or threonine in all species except shrew, where it is alanine. Substitution to a non-phosphorylatable residue would be of biological interest, but it is also possible that this position simply represents a genome assembly error in shrew. This position has *p_MLS_*=0.006, making it the least significant position of the five. Our method could exclude it and many similar cases by adding more criteria to the position selection process, but at the cost of decreased sensitivity and increased complexity. We expect that the coming availability of high-coverage *de novo* mammalian genome assemblies will resolve many such cases (e.g., [34], [35]).

F473 faces forward in our model. It is conserved hydrophobic (F or L) in all species except guinea pig, rat, mouse, and shrew, where it is S or C. Thus, F473 may be involved in a hydrophobic interaction with a binding partner.

We have low confidence in the exact placement of the short C-terminal loop, because it is not conserved in BMPR2 and lacks contacts with the other elements of our model. However, both E513 and H515 on this loop are preferentially charged in long-lived species, consistent with differential binding affinity. E513 is conserved in all species except rat, mouse, and shrew, where it is V, G, and A. H515 is conserved positive (H or R) in 16/17 species with MLS at least 16 y, but it is charged (H, R, or D) in only 3/9 shorter-lived species. The preference for hydrophilic residues in long-lived species at these two positions specifically may suggest that flexibility of the C-terminal loop is a longevity-conserved property.

Most of the residues on the αG–αH loop face the solvent, suggesting that they may interact with another domain or protein. If this is the case, then assuming that the gross function of AMHR2 is conserved within mammals, we would expect other residues on a common surface with the αG–αH loop to be conserved. We used positional conservation analysis to determine conservation of the surface residues of two alignments, (1) mammalian AMHR2 orthologs exclusively and (2) a representative set of mammalian orthologs in the TGFBR2 subfamily, including orthologs of TGFBR2, activin receptor type-2A and B (ACVR2A and ACVR2B), BMPR2, and AMHR2 (not shown). This analysis revealed two conserved solvent-facing patches flanking the region of our longevity-selected positions. One patch, mainly comprising the αH–αI loop, is conserved in all TGFBR2 subfamily members, confirming a previous observation [36]. The other patch, mainly comprising the αF–αG loop, is conserved only in AMHR2 orthologs.

Overall, these findings are consistent with the existence of a large interaction surface conserved in all AMHR2 orthologs whose area, and thus binding affinity, varies at the αG–αH loop in a species-specific manner. It is likely that this is a novel docking surface involved in the regulation of AMHR2; one possible regulatory binding partner is the C-terminal tail of AMHR2 itself, whose positions also have consistently low *p_MLS_* values relative to the proteome overall, as noted above. Since loop αG–αH flanks this conserved patch, and four of the five residues face solvent in our model, it is possible that overall these positions contribute to lifespan-specific binding affinity.

We are unaware of reported mutations specifically in the two loops of AMHR2 that contain our longevity-selected positions. However, two prior lines of inquiry are consistent with the docking-surface hypothesis. First, Belville et al. [36] also mapped the AMHR2 kinase domain to a solved structure in order to investigate natural mutations found in PMDS. Although based on a structure of the more distantly-related ACVR2B instead of BMPR2, its details are similar to those of our model, including both overall tertiary structure and specific residue orientation. Of the seven mutated positions they analyzed, four were located in the C-lobe of the kinase domain. One, D491, lies in the conserved αH–αI loop and faces solvent; its mutation to H causes PMDS. This mutation further supports the idea that the solvent-facing bottom of the C-lobe is critical for proper AMHR2 function.

Second, closer to the two loops on the bottom face of the domain is the residue E485, which is in αH and also faces solvent. In two independent population studies of Dutch women, the mutation E485Q was associated with menopause delayed by up to one year [37], [38]. In addition to its canonical role in male fetal development, AMHR2 also plays a second role in adult reproductive function. It is expressed in granulosa cells of adult females, where it seems to act as a feedback inhibitor of follicle recruitment and growth by binding its ligand, AMH, which is secreted in a paracrine manner specifically by more mature follicles [11]. Follicle depletion is the cause of menopause [1], and follicular decline or menopause has been observed in most or all mammals studied, including whales [39], [40], nonhuman primates [41], rodents, and others [1], [42], although admittedly we lack data for most species in the wild. A reasonable deduction is that a species’s rate of follicle depletion scales inversely with its longevity. We speculate that differential regulation of AMHR2 in a lifespan-dependent manner could act as a mechanism that effects this scaling, increasing the probability that a female has used all her reproductive potential before her death. This hypothesis could be viewed as a case of the disposable soma theory of aging [43]. It might be tested by relating AMHR2 ortholog sequence to rate of follicular decline across several species.

We also note the unusual cysteine conservation in the C-terminal tail, which is unique to AMHR2 orthologs. There are eight cysteine residues in this region of human AMHR2, all of which are relatively conserved. Multiple regression revealed a specific fit of the number of cysteines conserved to log_10_ MLS (*p_MLS_*=0.002 and *p_mass_=*0.082 after phylogenetic correction). It is possible that these residues bind zinc or another metal ion, thus imparting structure to this region, but the region does not match known zinc finger motifs.

### 3.5. Comparison with Previous Methods

Generally, we did not observe overlap between the longevity-selected proteins we identified and those identified in previous work [4], [5]. But this is not surprising, because we differed in our assumptions, goals, and data sets (detailed in Sections 1 and 2.1). Most notably, we fit a smaller subset of high-quality protein alignments, focused on structured protein regions, and chose to ignore synonymous codon substitutions. Thus, we view our results as complementary, not conflicting. We do note that both our method and that of Li and de Magalhães [5] identified ";myosin complex"; as an ontology term enriched in longevity-selected proteins (Table 3). The two methods also agreed that two proteins were longevity selected, rho guanine nucleotide exchange factor 16 (ARHGEF16) and lymphokine-activated killer T-cell-originated protein kinase (PBK). As both myosin and ARHGEF16 are involved in cell migration [44], there may be lifespan-specific differences in this activity, or our findings may simply reflect its standard role in organismal development.

One apparent novelty of our method is that it allows analysis of individual positions in the proteome, not just entire proteins. In fact, this is not novel, as the method of Jobson et al. [4] also entails identification of specific longevity-selected positions, and the genes they called ";longevity-selected"; and ";longevity-relaxed"; were simply genes with statistical over- or under-abundances of such positions. Here, we have emphasized individual positions rather than entire proteins for three reasons. First, we think it plausible, as did Jobson et al., that a mark of a longevity-selected protein is an abundance of longevity-selected positions. Second, a focus on individual positions allows to more precisely determine arbitrary regions of a protein that may be longevity-selected, as exemplified by the C-terminus of AMHR2. In theory, such specific focus can suggest novel biochemical mechanisms. Third, since aging is a complex trait that is under weak selection, it is plausible that some major determinants are subtle general properties of the proteome itself (discussed in Section 3.2), rather than the explicit activity of a single protein or even a few functional collections of proteins. Longevity-selected positions are the most obvious markers of such properties, and so may provide clues to identify them, in the same way as they may identify individual longevity-selected proteins. In short, we do not suggest that the positions that we call longevity-selected, in isolation, are major determinants of mammalian longevity. We only suggest that they may mark proteins or proteomic features that *are* such determinants, but are less obvious.

For detecting longevity-selected protein positions, benefits of our method versus standard codon-level methods such as the codeml program of PAML [45] include emphasis on detection of significant biochemical changes that are likely to affect protein structure; straightforward single-position resolution, allowing to easily test hypotheses regarding arbitrary regions of proteins, as described above; simple control for species-specific mutation rate and shared phylogeny and fitting of body mass as an alternate hypothesis to MLS; avoidance of the need to arbitrarily bin species by MLS; and faster run time. We reiterate that our method in theory is compatible with any quantitative trait, as illustrated by our inclusion of both MLS and body mass, although in practice correlations between predictor variables in the data limit the application of this for the predictors of greatest interest, such as developmental schedule (Section 3.3). However, this is a limitation that our approach shares with the others, given the set of species available. Some unique drawbacks of our method, in addition to those described in Section 3.4, include its reliance on a single reference proteome and lack of a specific model of protein evolution, implying unsuitability for rigorous statistical hypothesis-testing. It is a task for future work to combine the benefits of our method with those of standard codon model-based methods.

### 3.6. Conclusion

Based on principles of protein structure, we have developed a simple, extensible, and gerontologically-oriented method to find longevity-selected positions in the mammalian proteome. Using this method we found that, surprisingly, longevity-selected positions are much more common in the mammalian proteome than would be expected based on a randomized control. We have also used our method to identify specific protein regions that deserve further study in the context of the comparative biology of aging and development, as well as specific aging-related proteins that are likely not lifespan-conserved due to their overall conservation. Topping the list of regions worth further study is the kinase domain and C-terminal tail of AMHR2, in which the longevity-selected residues lie on a common surface of unknown functional significance. Our results must be considered in light of the flaws we described in the method, the high error and omission rates of the mammalian proteome data that we used, and most of all the obvious limitations inherent in using the set of putative orthologs from a single individual’s genome to represent the proteome of an entire species. Given those caveats, we have found our method to be a reasonable starting point for comparative analysis of protein function.

## Supporting Information

Figure S1 **Density histograms of** ***p_MLS_*** **values yielded by fitting alternate input sets with the method described in Section 2.2. As in** **Figure 3** **, only fit positions with** ***b_MLS_*** **>0 are included.** A. Chimp *(Pan troglodytes)* is the reference, and human is absent. B. Rhesus *(Macaca mulatta)* is the only primate fit. Human is the reference. C. Dog *(Canis lupus)* is the reference. D. Scores taken from BLOSUM62 instead of BLOSUM80. Real data. E. Randomized control data for (D).
(EPS)

Table S1 **The 107 protein domains that contain at least two longevity-selected positions.**
(XLS)

Table S2 **Longevity-selected positions of the protein domains shown in Table S1.**
(XLS)
